# Potential considerations in decision making on laparoscopic colorectal resections in Hungary based on administrative data

**DOI:** 10.1371/journal.pone.0257811

**Published:** 2021-09-27

**Authors:** Zsófia Benedek, Cecília Surján, Éva Belicza

**Affiliations:** 1 Mental Health Sciences Doctoral School, Semmelweis University, Budapest, Hungary; 2 Health Services Management Training Centre, Semmelweis University, Budapest, Hungary; Ohio State University Wexner Medical Center Department of Surgery, UNITED STATES

## Abstract

**Background:**

Laparoscopic colorectal surgeries offer numerous advantages over their open counterparts. To compare these measurable short-time outcomes of open and laparoscopic resections in Hungary, data of colorectal surgeries were collected and analysed. The study focused on identifying patients’ characteristics that can influence the decision on laparoscopic colorectal resections and on comparing efficiency of Hungarian colorectal operations with international data.

**Methods:**

Using patients’ data of laparoscopic and open colorectal surgery performed in 2015 and 2016 from the National Health Insurance Fund of Hungary, a countrywide retrospective comparative analysis was done. Logistic regression was used to explore main influencing factors for laparoscopic colorectal surgery.

**Results:**

A total of 17,876 colorectal surgical cases, including 14,876 open and 3,000 laparoscopic resections were selected and analysed. Laparoscopy was used only in 16.78% of all cases. Comparison of age groups showed that odds ratio (OR) of laparoscopic colorectal resections was significantly lower in over 40 years than in younger patients (18–39 years). In university institutes patients had higher odds (OR: 2.23 p<0.0001) for laparoscopic colorectal resections. Presence of comorbidity codes and preoperative treatment in internal medicine department decreased odds for laparoscopic colorectal operations.

**Conclusions:**

Patients’ age, comorbidities and hospital type influenced the likelihood of decision on laparoscopic colorectal resection. Selection of patients contributed to improved laparoscopic outcomes.

## Introduction

Laparoscopic colorectal resection (LCR) techniques offer numerous advantages over their open counterparts, including shorter hospitalization, faster recovery and reduced morbidity. Short-term physical benefits include decreased pulmonary and gastrointestinal morbidity, improved pain control, less dehiscence and surgical site infections, less postoperative hernia and blood loss [1–7]. There is no significant difference in oncological outcomes [6–11]. Laparoscopic resections are superior to open colorectal resections regarding physical functioning and vitality [12] and complication rates [6,13–15]. Studies have shown that laparoscopic resection was a safe and viable alternative to open colorectal surgery [7,16]. In Hungary, laparoscopic colorectal surgeries have been used since the 2000s. Nowadays, laparoscopic colorectal procedures have become a daily routine in many surgical departments. LCR were not clearly documented in the Hungarian healthcare database before 2014; surgeons could use only the intervention codes of open colorectal resections (OCR) at administration, and hospitals received the same funding both for laparoscopic and open surgeries [17]. Financial constraints decelerated the spread of modern laparoscopic colorectal procedures, and administrative deficiency caused untraceable data of LCR [18]. In February 2014 the codes were modified, and the health insurer started to finance the investment for special laparoscopic equipment. Since then LCR and OCR data can be compared from the National Health Insurance Fund of Hungary (Nemzeti Egészségbiztosítási Alapkezelő). Data transparency seems to be improved because identified and unique coded OCR and LCR cases can be followed up. The aim of our study is to identify independent predictors that influence the decision to use laparoscopic techniques for colorectal resections in Hungary.

## Methods

Using the National Health Insurance Fund Administration database, we have collected and analysed data of elective colorectal surgeries between January 1, 2015 and December 31, 2016. This study involves only anonymized administrative medical data. According to the Article 18 of Act XLVII of 1997 on the processing and protection of health care data and associated personal data, there is no need for the approval of the Research Ethics Committee for the analysis of administrative data requested from the National Healthcare Service Center (Állami Egészségügyi Ellátó Központ- AEEK). According to the above statement, the request and analysis of administrative data under the contract between Semmelweis University and the National Healthcare Service Center (registration number: ÁEEK/000810-001/2017) are not subject to the need for an ethical permission request. Elements of database were collected from 70 hospitals in Hungary, where colorectal surgeries were performed. Our research units were colorectal surgeries. In order to identify the type of colon or rectal procedures performed (Table 1), we used the Hungarian classification of procedures.

**Table 1:** Open and laparoscopic colorectal surgical procedures included in the study.

	Laparoscopic colorectal procedures	Open colorectal procedures
**Resection of the rectum**	Laparoscopic abdominoperineal resection of the rectum	Open abdominoperineal resection of the rectum
	Laparoscopic resection of the rectum with saving sphincter function	Open resection of the rectum with saving sphincter function
**Large bowel resection**	Laparoscopic sigmoidectomy or resection of rectosigmoid colon	Open sigmoidectomy or resection of rectosigmoid colon
	Right laparoscopic hemicolectomy	Open right hemicolectomy
	Laparoscopic partial colectomy	Open partial colectomy
	Left laparoscopic hemicolectomy	Open left hemicolectomy
	Laparoscopic colectomy	Open colectomy
	Laparoscopic proctocolectomy with ileostomy	Proctosigmoidectomy (Hartmann’s operation)

Procedure codes were associated with patient characteristics (age, sex, postoperative morbidity and comorbidity International classification of diseases (ICD) codes), 30-day mortality, and hospital identification code. ICD diagnoses were divided into two groups: main diagnosis (indicating the surgery) and secondary diagnoses (comorbidities and complications). The 70 inpatient hospitals involved, where colorectal surgeries were performed during the study time period were categorized by their type, and divided into three groups: university clinics and national centres, county centres and rural hospitals. Data of hospitalization (immediate before the operation) in department of internal medicine were also collected. Inclusion criteria were: age over 18 years, included open and laparoscopic colorectal surgical procedures (Table 1), elective surgical treatment, surgery performed between 1 January 2015 and 31 December 2016. Exclusion criteria were: inadequate main diagnosis (surgery-indicated morbidity, inadequate length of stay (number of days less than zero), length of hospital stay longer than 150 days and length of stay in the surgical department longer than 60 days until discharge or until transfer to a rehabilitation unit. After applying the inclusion and exclusion criteria, 17,876 colorectal surgical cases were categorized by SPSS into either LCR or OCR groups. 15,549 of main diagnoses were categorized into major main diagnosis groups (Table 2).

**Table 2:** Included diagnosis groups categorized by type.

Main diagnosis groups (Hungarian version of ICD codes)	Secondary diagnosis groups (Hungarian version of ICD codes)	
	Complications (in-hospital)	Comorbidities
Malignant neoplasm of colon (C18**-C19**)	Pneumonia and other acute lower respiratory infections (J12**-22**)	Secondary malignant neoplasm of liver and intrahepatic bile duct (C7870)
Malignant neoplasm of rectum (C20H0)	Abscess, perforation, fistula, and ulcer of intestine (K631*-K633*)	Essential (primary) hypertension (I10H0)
Benign tumours of colon and rectum (D12**)	Peritonitis (K65**)	Hypertensive heart disease, Chronic ischaemic heart disease (I11*, I25**)
Neoplasms of uncertain behaviour of colon and rectum (D3740-D3750)	Cutaneous abscess, furuncle and carbuncle and pressure ulcer (L023*,L023*, L89**)	Peritoneal adhesions (K66**)
Crohn’s disease, large intestine (K5010-K5190)	Acute posthaemorrhagic anaemia, and anaemia, unspecified (D62**, D649*)	Symptoms and signs concerning food and fluid intake (R6330)
Diverticular disease of large intestine without perforation or abscess (K5730, K5750, K5790)	Disorders of electrolyte intake and/or acid base balance (E87**)	Chronic obstructive pulmonary disease (COPD) (J44**)
	Paralytic ileus and intestinal obstruction without hernia (K56**)	Atrial fibrillation and flutter (I48**)
	Acute renal failure(N17**)	Non-insulin-dependent diabetes mellitus (E11**)
	Complications of procedures, not elsewhere classified (T81**)	Atherosclerosis (I70**)
	Acute pulmonary insufficiency following nonthoracic surgery, respiratory failure, not classified elsewhere (J952*, J96**)	Chronic renal failure (N18**)
	Other respiratory disorders (J98**)	
	Septicaemia (A40**-A41**)	

*or **: Third or fourth variable values were marked with * and ** in diagnosis codes, as diagnosis groups were used in the study.

To determine factors influencing surgical decision-making for open versus laparoscopic colorectal procedures, multivariable logistic regression analysis was performed. Odds ratio (OR) with a 95% confidence interval was calculated to determine the combined effect of various preoperative factors for laparoscopic colon and rectal operations. These variables included patient age and sex, comorbidities (Table 2), main diagnosis groups (Table 2), and hospital type. To determine factors influencing 1–30 day unadjusted postoperative mortality, a second multivariate logistic regression was performed. OR with a 95% confidence interval was calculated. Mortality was included as the dependent variable and surgical procedure (OCR and LCR groups), patient age, comorbidities (Table 2), main diagnosis groups (Table 2), and complications (Table 2) were independent factors. Age categories and surgical procedures were categorical, and diagnoses were binary variables. A statistical significance level was defined at alpha <0.05. All statistical analyses for the database were conducted using SPSS (25^th^ version).

## Results

Of the 17,876 colorectal resections that met inclusion criteria, 14,876 were open and 3,000 were laparoscopic. Overall, laparoscopy was used only in 16.78% of all colorectal cases. Mean age was 66.35 (SD: 12.68) years in the OCR group and 63.78 (SD: 12.62) years in the LCR group (p<0.0001). Distribution of sexes was statistically not significant between the two groups (p = 0.124) with Pearson Chi^2^ test (χ^2^(1) = 2,363; p = 0,124). Patient sex and age data for the laparoscopic and open groups are given in Table 3.

**Table 3:** Distribution of laparoscopic and open colorectal surgeries by sex and age.

Cross-tabulation of sex, age categories of LCR and OCR				
		Surgery type		
	age	OCR (number of cases)	LCR (number of cases)	Total_(number of cases)_ = LCR+OCR and (LCR% of Total)
**male**	18–39	331	69	400 (LCR: 17.3%)
	40–49	428	109	537 (LCR: 20.3%)
	50–59	1,125	256	1381 (LCR: 18.5%)
	60–69	2,804	625	3,429 (LCR: 18.2%)
	70–79	2,495	437	2,932 (LCR: 14.9%)
	80-x	954	99	1,053 (LCR: 9.4%)
	Total	8,137	1,595	9,732 (LCR: 16.3%)
**female**	18–39	230	91	321 (LCR: 28.4%)
	40–49	328	122	450 (LCR: 27.1%)
	50–59	928	221	1,149 (LCR: 19.2%)
	60–69	1,975	467	2,442 (LCR: 19.1%)
	70–79	2,114	379	2,493 (LCR: 15.2%)
	80-x	1,164	125	1,289 (LCR: 9.7%)
	Total	6,739	1,405	8,144 (LCR: 17.3%)

In the study population, it seemed that the rate of LCR cases decreased in both sexes over the age of 70 (LCR% was between 14.9% and 9.4%). Although there was a low incidence of colorectal resection in women between the age of 18–39 and 40–49, this group had the highest proportion of LCR (28.4% and 27.1% of all operated cases, respectively). Distribution of main diagnosis groups was analysed (Table 4) in context of LCR, OCR groups and sex.

**Table 4:** Main diagnoses indicating surgery in laparoscopic and open surgery groups.

			Malignant neoplasm of colon	Malignant neoplasm of rectum	Benign neoplasm of colon and rectum	Neoplasm of uncertain behaviour of colon and rectum	Crohn’s disease, large intestine	Diverticular disease of large intestine without perforation or abscess	Total (N)
**Male**	**OCR**	**(N)**	4,213	1,934	158	309	290	170	7,074
	**LCR**	**(N)**	767	521	84	86	49	58	1,565
		**LCR%**	15.4	21.2	34.7	21.8	14.5	25.4	18.1
**Female**	**OCR**	**(N)**	3,380	1,195	123	327	254	263	5,542
	**LCR**	**(N)**	709	335	93	92	61	78	1,368
		**LCR%**	17.3	21.9	43.1	22	19.4	22.9	19.8
**Total**		**(N)**	9,069	3,985	458	814	654	569	15,549
		**LCR%**	16.3	21.5	38.6	21.9	16.8	23.90	18.9

LCR% = Number of cases LCR/(Number of cases OCR+LCR)*100, (N) = number of cases in the main diagnosis groups of Table 4.

The most common indication for surgery was malignant neoplasm of the colon (58.3%), of which 16.3% were performed laparoscopically. The condition with the highest proportion of patients receiving LCR was diverticulosis, with a rate of 23.9%. Colon and rectal malignant tumours were the most frequent reasons in men for open and laparoscopic procedures as well (4,213 vs. 767 cases). We found some distributional differences of main diagnoses regarding open and laparoscopic surgeries in the two sexes. The rate of LCR in cases of female patients was higher than in male patients in all (main) diagnoses indicating the surgery, except for diverticulosis: male with 25.4% vs. female with 22.9%. (Table 4). 40.2% (1,206 of 3,000) of LCR were performed in university or national institutes, and 35.5% (5281 of 14,876) of OCR were performed in county centre hospitals. Rate of LCR technique was 4.4% (780 of 17,876) in rural, 5.7% (1,014 of 17,876) in county hospitals, and 6.7% in university and national institutes (1,206 of 17,876).

Characteristics were analysed as independent risk factors influencing the decision on the LCR technique in multivariable forward logistic regression analysis (Table 5).

**Table 5:** Variables influencing the choice of LCR by multiple logistic regression.

	Variables	Odds ratio (OR)	95% C.I.	p value
	Age category (Ref.: 18–39 years)			p<0.0001
	40–49 years	0.844	(0.650–1.095)	p = 0.202
	50–59 years	0.644	(0.504–0.821)	p<0.0001
	60–69 years	0.633	(0.500–0.800)	p<0.0001
	70–79 years	0.555	(0.437–0.706)	p<0.0001
	80-x years	0.403	(0.342–0.515)	p<0.0001
	Female (Ref.: Male)	1.128	(1.038–1.226)	p = 0.004
	Preoperative treatment in department of internal medicine	0.420	(0.342–0.515)	p<0.0001
**Main diagnoses**	Malignant neoplasm of colon	6.382	(4.954–8.221)	p<0.0001
	Malignant neoplasm of rectum	7.597	(5.864–9.842)	p<0.0001
	Benign neoplasm of colon and rectum	17.651	(12.886–24.177)	p<0.0001
	Neoplasm of uncertain behaviour of colon and rectum	8.695	(6.442–11.737)	p<0.0001
	Crohn’s disease, large intestine	3.068	(2.174–4.330)	p<0.0001
	Diverticular disease of large intestine without perforation or abscess	8.265	(6.026–11.336)	p<0.0001
**Secondary diagnoses**	Secondary malignant neoplasm of liver and intrahepatic bile duct	0.420	(0.329–0.538)	p<0.0001
	Essential (primary) hypertension	0.847	(0.772–0.929)	p<0.0001
	Hypertensive heart disease or chronic ischaemic heart disease	0.755	(0.663–0.861)	p<0.0001
	Peritoneal adhesions	0.683	(0.542–0.861)	p = 0.001
	Symptoms and signs concerning food and fluid intake	0.779	(0.596–1.022)	p = 0.071
	Chronic obstructive pulmonary disease	0.596	(0.441–0.805)	p = 0.001
	Atrial fibrillation and flutter	0.687	(0.547–0.863)	p = 0.001
	Diabetes mellitus	0.918	(0.794–1.062)	p = 0.251
	Atherosclerosis	0.556	(0.414–0.745)	p<0.0001
	Chronic renal failure	0.383	(0.845–1.233)	p = 1.233
	Hospital type (Ref.: Rural)			p<0.0001
	County centre	1.019	(0.918–1.130)	p = 0.728
	University or national centre	2.229	(2.0006–2.476)	p<0.0001
	Number of cases (n)	17,876		

Age categories, sex, main diagnoses, majority of secondary diagnoses, and university or national centres were significantly associated with choosing LCR technique over OCR (Table 5). Multivariable logistic regression (Table 5) showed that the chance of patients being selected for LCR progressively decreased over the age of 40. Over 80 years OR was only 0.4 (p<0.0001). Table 5 highlights differences of OR of main diagnoses. OR was over 6 in any type of neoplasms, with the highest value in benign colorectal tumours (OR: 17.65; p<0.0001). Overall, we found that women had a slightly higher chance for laparoscopic colorectal surgery (OR: 1.13; p = 0.004). Logistic regression model demonstrated that patients with comorbidity diagnoses in their medical records (e.g. secondary malignant neoplasm of liver and intrahepatic bile duct, peritoneal adhesions, COPD, atrial fibrillation, atherosclerosis, hypertensive heart disease and chronic ischaemic heart disease) had reduced likelihood for LCR. Regarding comorbidity diagnoses, preoperative treatment in internal care units seemed to decrease the probability of laparoscopic colorectal surgery (OR: 0.42; p<0.0001). Considering the type of operating institutes, we found high odds ratio (OR: 2.23; p<0.0001) for LCR in university and national institutes compared to rural and county centre hospitals.

We collected and categorized complications into diagnosis groups by frequency (Table 2). 36% of open and 3.1% of laparoscopic colorectal procedures were associated with a minimum of one complication code. We found that postoperative (unadjusted) mortality within 1 to 30 days was considerably higher if open surgery was performed (8.2% and 1.2% in the OCR and LCR group, respectively). To show the effect of LCR procedure on 1–30 day postoperative mortality- as it was recommended- we made a second multivariate logistic regression, where mortality was dependent and surgical procedures were presented as independent risk factors (Table 6). After checking multicollinearity, we had to exclude some of our previous variables like hospital types or comorbidity (e.g. COPD), or patient sex.

**Table 6:** Variables influencing unadjusted postoperative mortality by Multiple Logistic Regression.

Variables			Odds ratio (OR)	95% C.I.	p value
		Age category (Ref.: 18–39 years)	Ref		p<0.0001
		40–49 years	2.854	(1.209–6.739)	p = 0.017
		50–59 years	4.751	(2.199–10.265)	p<0.0001
		60–69 years	6.745	(3.180–14.307)	p<0.0001
		70–79 years	11.785	(5.555–25.004)	p<0.0001
		80-x years	25.347	(11.889–54.041)	p<0.0001
		Surgery (Ref.: Opened)	0.326	(0.226–0.463)	p<0.0001
**Main diagnoses**		Malignant neoplasm of colon	0.452	(0.381–0.537)	p<0.0001
		Malignant neoplasm of rectum	0.306	(0.239–0.391)	p<0.0001
		Benign neoplasm of colon and rectum	0.204	(0.097–0.428)	p<0.0001
		Neoplasm of uncertain behaviour of colon and rectum	0.506	(0.348–0.736)	p<0.0001
		Crohn’s disease, large intestine	0.542	(0.284–1.035)	p = 0.071
		Diverticular disease of large intestine without perforation or abscess	0.322	(0.187–0.556)	p<0.0001
**Secondary diagnoses**	**Comorbidities**	Secondary malignant neoplasm of liver and intrahepatic bile duct	2.914	(2.319–3.662)	p<0.0001
		Essential (primary) hypertension	0.829	(0.716–0.961)	p = 0.013
		Hypertensive heart disease or chronic ischaemic heart disease	1.271	(1.086–1.487)	p = 0.003
		Peritoneal adhesions	0.666	(0.497–0.893)	p = 0.007
		Symptoms and signs concerning food and fluid intake	0.204	(0.099–0.420)	p<0.0001
		Atrial fibrillation and flutter	1.246	(1.015–1.530)	p = 0.035
		Diabetes mellitus	1.245	(1.026–1.511)	p = 0.027
		Atherosclerosis	2.073	(1.690–2.543)	p<0.0001
		Chronic renal failure	2.012	(1.515–2.672)	p<0.0001
	**Complications**	Pneumonia and other acute lower respiratory infections	2.206	(1.665–2.922)	p<0.0001
		Peritonitis	2.336	(1.934–2.823)	p<0.0001
		Cutaneous abscess, furuncle and carbuncle and pressure ulcer	0.506	(0.352–0.728)	p<0.0001
		Acute posthaemorrhagic anaemia, and anaemia, unspecified	1.398	(1.189–1.622)	p<0.0001
		Disorders of electrolyte intake and/or acid base balance	0.801	(0.671–0.956)	p = 0.014
		Paralytic ileus and intestinal obstruction without hernia	1.624	(1.357–1.944)	p<0.0001
		Acute renal failure	2.720	(2.042–3.624)	p<0.0001
		Acute pulmonary insufficiency following nonthoracic surgery, respiratory failure, not classified elsewhere	2.631	(2.076–3.334)	p<0.0001
		Other respiratory disorders	2.156	(1.725–2.694)	p<0.0001
		Septicaemia	3.710	(2.995–4.595)	p<0.0001
		Number of cases (n)	17,876		

The multivariate logistic regression (Table 6) showed that having LCR procedure reduced likelihood for the 1–30 day postoperative death, if OCR group was the reference (OR: 0.326; p<0.0001). The mortality of patients progressively increased with age. Complications involved in regression (Table 6) are associated with elevated odds for mortality, the highest value belonged to septicaemia (OR: 3.710; p<0.0001). Acute renal failure (OR: 2.720; p<0.0001) and respiratory insufficiency (OR: 2.631; p<0.0001) or respiratory disorders (OR: 2.156; p<0.0001) showed also high OR values, their presence in medical records more than doubling odds of mortality. Our second regression model demonstrated that patients with comorbidity diagnosis in their medical records e.g. secondary malignant neoplasm of liver and intrahepatic bile duct, atherosclerosis and chronic renal failure diagnoses had an improved likelihood for mortality with the highest OR values. The elective surgical main diagnoses in this regression had a reduced likelihood for mortality as an independent predictor. The lowest OR value (OR: 0.204; p<0.0001) is connected to benign neoplasm of the colon and rectum.

## Discussion

In Hungary, the rate of laparoscopic colorectal resection among all elective colorectal surgery was only 16.7% in 2015–2016. Our study indicates that laparoscopic colorectal surgery was underused in Hungary, especially, if compared with international reports. The Surgical Care and Outcomes Assessment Program (SCOAP) evaluated the use of laparoscopy for elective colorectal resections in 48 hospitals in the United States in 2010, and a rate of 41.6% was found [19]. Askari et al reported that the percentage of laparoscopic colorectal resections was 20,9% in England between 2001 and 2011 [20]. We share the opinion of the National Institute for Health and Clinical Excellence (NICE) [21] that the limiting factor for the implementation of LCR is the number of surgeons capable of performing laparoscopic colorectal resections, rather than the characteristics of the tumour or the patient. We complement this opinion with the fact that financial constraints decelerated the spread of modern laparoscopic colorectal procedures in Hungary before 2014 [18]. If the increasing efficiency were aimed at, it would be useful to utilize the positive effect of a standardized surgical technique, training courses and surgical simulation on the implementation of laparoscopic colorectal procedures [22,23].

The most important findings of our study are the relationship between age, comorbidities, main diagnoses, type of operating hospitals and laparoscopic colorectal procedures (Table 5). Hungarian and international experiences [24–26] show a selection of healthier and younger patients for LCR during the learning period of laparoscopic colorectal surgery. Pascual reported that the number of absolute contraindications of LCR is currently almost negligible [27]. She found that appropriate patient selection is important to maintain conversion rates below 10%. We hypothesize that these selections played a role in better outcomes at laparoscopic colorectal resections, and should carefully planning methods (e.g.: randomized methods of sample groups) to analyse LCR and OCR outcomes or institutional results with other hospitals outcomes. The result of our second logistic regression (Table 6) confirms that having LCR against OCR and well-chosen indications had positive effect on unadjusted postoperative mortality. To analyse output data clearly, it would be necessary to standardize surgical indication for LCR and OCR. The standardized procedure could help to establish indicators of colorectal surgical quality like MTL30 [28]. In our database it seems that women had slightly higher odds for laparoscopic colorectal resections (OR: 1.128; p = 0.004) than men. In the logistic regression, the OR pattern of age, presence of comorbidities and existence of preoperative medical treatment in internal medical departments suggested that older and multimorbid (more than one comorbidity) patients had less chance for LCR.

We found that the most frequent main diagnosis codes were malignant colorectal tumours (Table 4). These results can be related to the composition of indication of elective surgical cases and the high prevalence of colorectal cancer in Hungary [29]. Unfortunately, we could not use the data of preoperative staging survey in our study, as this information is not reported accurately in the database of the National Health Insurance Fund of Hungary. In our research, hospital types were factors associated with the laparoscopic colorectal resections as mentioned in SCOAP [19] or by Kemp [26] and Pascual [27]. In Hungary, universities or national institutes are more likely to be predictive factors for laparoscopic colorectal procedures (OR: 2.23; p<0.0001). We are convinced that it is related to education and technical possibilities in addition to financial support.

We noticed some limitation of our research at the beginning. We found a shockingly high rate of postoperative 1-30-day mortality (8.2%) in the OCR group, especially, if compared with 1.28% 30-day–mortality rate reported by Kellers nationwide study [4] or 2% in the COLOR II findings [30], or 1.4% in the Nationwide Inpatient Sample (NIS) database study [31]. We could not find any failure in our database or calculations. Mortality data could be influenced by a generally bad health status [29] of middle-aged and elderly Hungarian patients, as well as the lack of primary prevention of colorectal diseases and of the colonoscopy screening for colorectal tumours or quality of postoperative care and surgical experience. Our study implies that there was patient selection for LCR which could positively affect the outcome such as mortality or reoperation. To date, there is no specific procedure code for conversion from laparoscopic resection to open surgery, thus, our procedure code system does not allow quantifying number of conversions and early (on the same day of operation) reoperations. Surgical reports by emergency classification can be potentially inaccurate in OCR too. Therefore, our results should be interpreted critically.

The authors are confident that the present research will support the improvement of insurance data transparency and the development of patient care.
